# A method for modifying carbonaceous materials with manganese carbonate to module the formation of singlet oxygen via activation with Peroxymonosulfate

**DOI:** 10.1016/j.mex.2025.103252

**Published:** 2025-03-13

**Authors:** Camila Mosquera-Olano, Carolina Quimbaya, Santiago López-Pérez, Elkin Castellón-Castrillón, Sandra Navarro, John Rojas, Jorge Acosta, Ricardo A. Torres-Palma, Yenny P. Ávila-Torres

**Affiliations:** a Grupo de Investigación en Remediación Ambiental y Biocatálisis (GIRAB), Instituto de Química, Facultad de Ciencias Exactas y Naturales, Universidad de Antioquia UdeA, Calle 70 No. 52-21, Medellín, Colombia; b Grupo de Investigación Cecoltec, Cecoltec Services, Cra 43 A 18 sur 135, Medellín, Colombia; c People´s Friendship University of Russia (RUDN University), 6 Miklukho- Maklaya Street, 117198, Moscow, Russian Federation

**Keywords:** Basic medium, Oxidant agent, Acetaminophen, Degradation, A method for modifying carbonaceous materials with manganese carbonate to module the formation of singlet oxygen via activation with peroxymonosulfate

## Abstract

This approach examines several agriculture wastes (shell coconut, pseudo-steam banana, potato) as precursors of carbonaceous materials to produce ^1^O_2_. The materials were modified with manganese carbonate at 800 °C and then, characterized using DLS (Dynamic Laser Scattering), BET (Brunauer-Emmett-Teller) Method, and zeta potential. Subsequently, the materials were activated via-peroxymonosulfate (PMS, HSO_5_), and a scavenger to evidence the singlet oxygen formation was used. Acetaminophen, sulphamethoxazole, and losartan were employed as the pharmaceutical targets, evaluating the effects of ROS (reactive oxygen species) in the acetaminophen degradation processes. The methodology of computational calculations was developed to obtain HOMO-LUMO for all systems of modified and unmodified materials in the presence of PMS and acetaminophen to understand both the adsorption and the carbocatalysis processes. This method allows an understanding of the presence of manganese carbonate in thermal synthesis, which determines the modulation of reactive oxygen species depending on the pH of the treatment. This method is significant as follows:

• Low-cost carbonaceous materials activated with PMS demonstrated significant efficiency in degrading organic molecules, utilizing singlet oxygen as the primary oxidant.
• Theoretical computational approaches investigate the interaction between material and molecule.
• The role of carbonate ions in influencing singlet oxygen production in materials derived from residual biomass was examined.

## Graphical abstract

**Figure:**
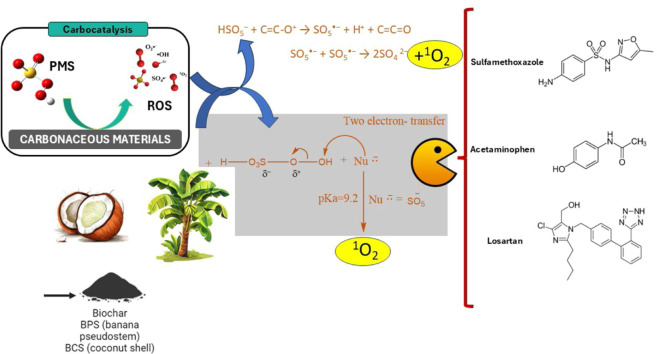

Specifications table

**Table:** 

Subject area:	Environmental Science
More specific subject area:	Pollutants degradation
Name of your method:	A method for modifying carbonaceous materials with manganese carbonate to module the formation of singlet oxygen via activation with peroxymonosulfate
Name and reference of the original method:	Carolina Quimbaya-Ñañez, Efraim A. Serna-Galvis, Javier Silva-Agredo, Lázaro Huerta, Ricardo A. Torres-Palma, Yenny Ávila-Torres, Mn-based material derived from industrial sawdust for the elimination of ciprofloxacin: Loss of antibiotic activity and toxicity via carbocatalysis assisted by ultrasound, Journal of Environmental Chemical Engineering, Volume 12, Issue 2, 2024, 112,015, https://doi.org/10.1016/j.jece.2024.112015.
Resource availability:	All resources are detailed in this article*.*

## Background

The ¹O₂ species has become increasingly significant, having major effects on emerging contaminants that have been clearly understood recently. This species shows a particular selectivity for heterocycle compounds containing sulfur, selenium, phosphorus, and some iridium transition metal complexes [1]. The interaction of ¹O₂ with such compounds may involve the formation of covalent adducts that transfer one or two oxygen atoms to the heteroatom center. Given the nature of these atoms, particularly when stereogenic centers are present, singlet oxygen plays an active role in stereoselective reactions. It has been noted that ¹O₂ exists just 22.5 kcal/mol above the ground state triplet, and its lifespan exceeds that of highly reactive free radicals such as hydroxyl, allowing this species to participate in chemical reactions in aqueous environments [2]. ¹O₂ reacts with emerging contaminants via electrophilic attacks on alkenes, oxygen addition, hydrogen elimination, and concerted Diels-Alder processes in cycloadditions, among others [3]. Remarkably, it also plays a role in reactions mediated by protic solvents, particularly in stabilizing sulfones, sulfur intermediates, disulfides, and sulfonamides, as well as attacking furans present in various pharmaceutical products. Further, due to its standard redox potential of 1.52 V, it can be detected using a quencher such as sodium aside (NaN_3_) with a reaction rate of 1 × 10^9^ M^−1^s^−1^. On the other hand, the evolution of ^1^O_2_ has been studied when PMS (peroxymonosulfate) was activated by quinones, phenols, alkalis, etc. These functional groups are present in biochar, providing an economical option to produce this non-radical species, Eqs. (1)–(2) [4,5].

(1) $$SO_{5\;}^{-}+C=C-O^{+}\to SO_{5}^{\cdot -}+H^{+}+C=C=O$$

(2) $$SO_{5\;}^{-}+SO_{5}^{\cdot -}\to 2SO_{4\;}^{2-\;}+{}^{1}O_{2}$$

Interestingly, the effect of buffering ions to control pH on producing reactive oxygen species is also considerable. Thus, for the generation of the sulfate anion radical (Eq. (3)) and singlet oxygen from PMS (Eqs. (4)-(2)), carbonate decreases the solubility of the metals incorporated in the carbonaceous matrix and facilitates the cleavage and their oxidation [6,7]

(3) $$\equiv M\left(\text{II}\right)\;+{\;\text{HSO}}_{5}^{-}\;\to \;\equiv M\left(\text{III}\right)\;+{\;\text{SO}}_{4}^{•-}\;+\;OH^{-}$$

(4) $$\equiv M\left(\text{III}\right)+{\;\text{HSO}}_{5}^{-}\;\to \;\equiv M\left(\text{II}\right)+{\;\;\text{SO}}_{5}^{•-}\;+\;H^{+}$$

However, carbonate also favors the HSO_5_^−^ / SO_5_^2^ equilibrium at a basic pH of around 9.4, (Eqs. (5)-(6)) resulting in higher production of this species as compared to radical species [8,9].

(5) $${\text{HSO}}_{5}^{-}\;\to H^{+}+{\;\text{SO}}_{5}^{-},\;\text{pka}=9.4$$

(6) $$HSO_{5\;}^{-}+\;SO_{5\;}^{2-\;}\to HSO_{4\;}^{-\;}+\;{}^{1}O_{2\;}$$

This study presents a method for producing modified carbonaceous matrices derived from coconut, banana pseudostem, and potatoes, using manganese carbonate as a precursor. The approach enables the generation of singlet oxygen in higher proportions compared to radical species such as sulfate radical anions and hydroxyl radicals. The production mechanism of singlet oxygen involves the pentoxide anion radical and the HSO_5_⁻/SO_5_⁻ equilibrium, both of which are enhanced by the presence of manganese in the structure. Manganese facilitates peroxide bond cleavage in the former mechanism and promotes deprotonation under basic pH in the latter. Consequently, this method highlights the role of carbonate incorporated into the material during synthesis, which promotes the nucleophilic attack of PMS on HSO_5_⁻ and facilitates singlet oxygen formation at elevated pH levels. In this context, residual biomass was subjected to pyrolysis at 500 °C, followed by modification with manganese carbonate and thermal exfoliation at 800 °C, leading to materials that can stabilize carbonates on their surface and promote basic equilibria around the pKa of the PMS molecule. This approach boosts the generation of singlet oxygen and facilitates the degradation of acetaminophen, losartan, and sulfamethoxazole.

## Method details

### Reagents

Merck provided MnCO_3_ and sodium aside (NaN_3_). OXONE® (KHSO_5_ 0.5KHSO_4_ 0.5K_2_SO_4_) was used as a source of potassium peroxymonosulfate (KHSO_5_) analytical degree. Acetaminophen (ACE), sulphamethoxazole (SFM), and losartan (LOS) were obtained from Abbott pharmaceutical laboratories, with an analytical degree.

## Reaction systems

The carbocatalysis experiments were performed in a batch-type reactor flocculation system, containing 500 mL of sample. Acetaminophen (ACE, 30.6 µM) was added to 0.5 mM of PMS and 0.2 g L^−1^ of the catalyst BPS (material from banana pseudo-stem), BPP (material from peel potato) or BSC (material from coconut shell) and its modifications with manganese was added. The catalyst and PMS concentrations were chosen according to previous work performed by our research team [8]. Adsorption and thermic exfoliation were carried out as controls for each carbon catalyst. The same procedure was used with sulphamethoxazole and losartan.

## Analyses

The evolution of ACE, SFM, and LOS concentration during the experiments was determined using a UHPLC Thermo-Scientific (Waltham, MA, USA) Dionex UltiMate 3000 instrument equipped with an Acclaim™ 120 RP C18 column (5 µm, 4.6 × 150 mm). For the chromatographic analysis of ACE, a mixture of acetonitrile and formic acid in a ratio of 30:70 (% v/v) and a flow of 0.5 mL min^−1^ was used, and the detection wavelength was set at 230 nm. For the chromatographic analysis of SFM, a mixture of acetonitrile and formic acid in a ratio of 85:15 (% v/v) and a flow of 0.5 mL min^−1^ was used, and the detection and the detection wavelength were set at 230 nm. For the chromatographic analysis of LOS, a mixture of methanol, acetonitrile, and formic acid in a ratio of 10:40:50 (% v/v) and a flow of 0.5 mL min^−1^ was used, and the detection and the detection wavelength were set at 280 nm.

## Spectroscopic characterization for (BPS, BPP, BCS, BPS-Mn, BPP-Mn, BCS-Mn)

The Brunauer–Emmett–Teller (BET) method was applied to measure samples' surface area and pore characteristics, utilizing a Quatachrome NOVA© 1200e analyzer (Boynton Beach, FL, USA). Particle size was determined through the Dynamic Light Scattering (DLS) analysis using a Malvern Zetasizer Pro (Malvern, UK).

## Computational calculations

Computational modeling was performed to gain insight into the adsorption interactions between carbon materials and only acetaminophen compounds. This simulation study was conducted to evaluate the interaction and adsorption of key molecules, such as acetaminophen (protonated and deprotonated forms on the oxygen ring) and PMS (peroxymonosulfate, in both protonated and deprotonated forms), on two types of activated carbon surfaces: (i) one obtained by pyrolysis and (ii) another modified with Mn (manganese). The calculations were divided into two phases, described in this method [10]:

**Phase 1: Energy Optimization:** Individual molecules (activated carbon, Mn-coordinated activated carbon, acetaminophen, and PMS in their protonated and deprotonated forms) underwent energy optimization using the Dmol3 module of Materials Studio software. This step was crucial for obtaining minimum-energy geometries, which would later be used in the adsorption calculations. Calculation Details: The GGA (Generalized Gradient Approximation) function was employed with the PBE (Perdew-Burke-Ernzerhof) function. This approach is well-suited for systems with Van der Waals interactions, as GGA considers variations in electron density, while PBE is known for high accuracy in coordination and surface systems, balancing precision, and efficiency. Basis Set: DND (Double Numerical plus Polarization), selected to accurately describe bonds and partial charges in complex systems, such as Mn-coordinated activated carbon and organic structures. Basis File: 4.4, an updated and optimized basis file for high-precision simulations in the Materials Studio environment. Convergence Tolerances: Energy: 2.0e-5 Ha, ensuring high precision in energy minimization for stable configurations. Maximum Force: 0.004 Ha/Å, providing precision for internal atomic forces to ensure the obtained geometry is near minimum energy. Maximum Displacement: 0.005 Å, minimizing atomic position shifts for reliable geometries without significant structural distortion. Energy optimization for each molecule sets the foundation for accurate adsorption analysis in the next phase.

**Phase 2: Adsorption Calculation:** In the second phase, the Adsorption module in Materials Studio software BIOVIA 2019 19.1, was used to calculate and evaluate various aspects of adsorption energy, such as rigid adsorption energy, deformation energy, and the total energy of the systems formed between adsorbates (acetaminophen, deprotonated acetaminophen, PMS, deprotonated PMS) and the two types of activated carbon.

**Procedure and Parameters: The adsorption phase was divided into two steps:**

(i) **Adsorption on Pyrolyzed Activated Carbon:** The adsorption of each adsorbate on pyrolyzed activated carbon was studied, considering both individual and combined interactions. Each calculation involved positioning each adsorbate on the activated carbon surface, starting with individual adsorptions, and then combining adsorbate molecules to create a system with activated carbon and all adsorbates. Calculations were performed at distances of 1 Å and 5 Å to compare the effects of proximity on adsorption energy.
  Adsorption on Mn-Coordinated Activated Carbon: The same adsorption procedure was repeated on the Mn-coordinated activated carbon structure. Mn presence plays a significant role in adsorption and potential electron transfer, highlighting differences in adsorption energy relative to non-coordinated activated carbon.
(ii) **Calculation Parameters:** Simulated Annealing was used to explore multiple configurations and ensure the system is at its lowest energy state. Quality: ultra-fine, selected for accuracy in energetic and geometric configurations. Force Field: COMPASSIII, a general force field suitable for organic and inorganic systems, enabling precise intra- and intermolecular interaction calculations. Charges: Assigned by the force field to accurately consider partial charges and reflect system polarization. Low-Energy Configurations: A set of 15 low-energy configurations was calculated, maintaining the original structure of the substrate and adsorbates. This allowed analysis of how specific adsorbate interactions affect the geometry and adsorption energy in the system. The results of this phase reveal how each adsorption system (activated carbon or Mn-coordinated activated carbon) interacts with adsorbed molecules, providing detailed insight into the affinity of each adsorbate for the surface.

Localized orbitals represent the spatial distribution of electrons within a molecule, crucial for understanding regions with higher electron density and their relation to active centers. These calculations allow us to visualize frontier molecular orbitals (HOMO and LUMO) and their locations in each molecule, offering a detailed view of potential interaction sites. Localized orbitals help interpret the molecule's reactivity and its adsorption or electron transfer potential when in contact with activated carbon.

## Method validation

### Carbonaceous material preparation

Banana pseudo-stem was collected from the local agricultural sector in Urabá, Colombia (Banaexport S.A.). Peel potato (PP) was collected from Pasto (Nariño, Colombia). The coconut shell was collected from the Pacificoco S.A.S. industry (Valle del Cauca, Colombia). The material was dried at room temperature for 24 h, ground using a manual mill, and separated by particle size. The fine powder that passed through mesh No. 40 was selected.

**Fig. 1:**
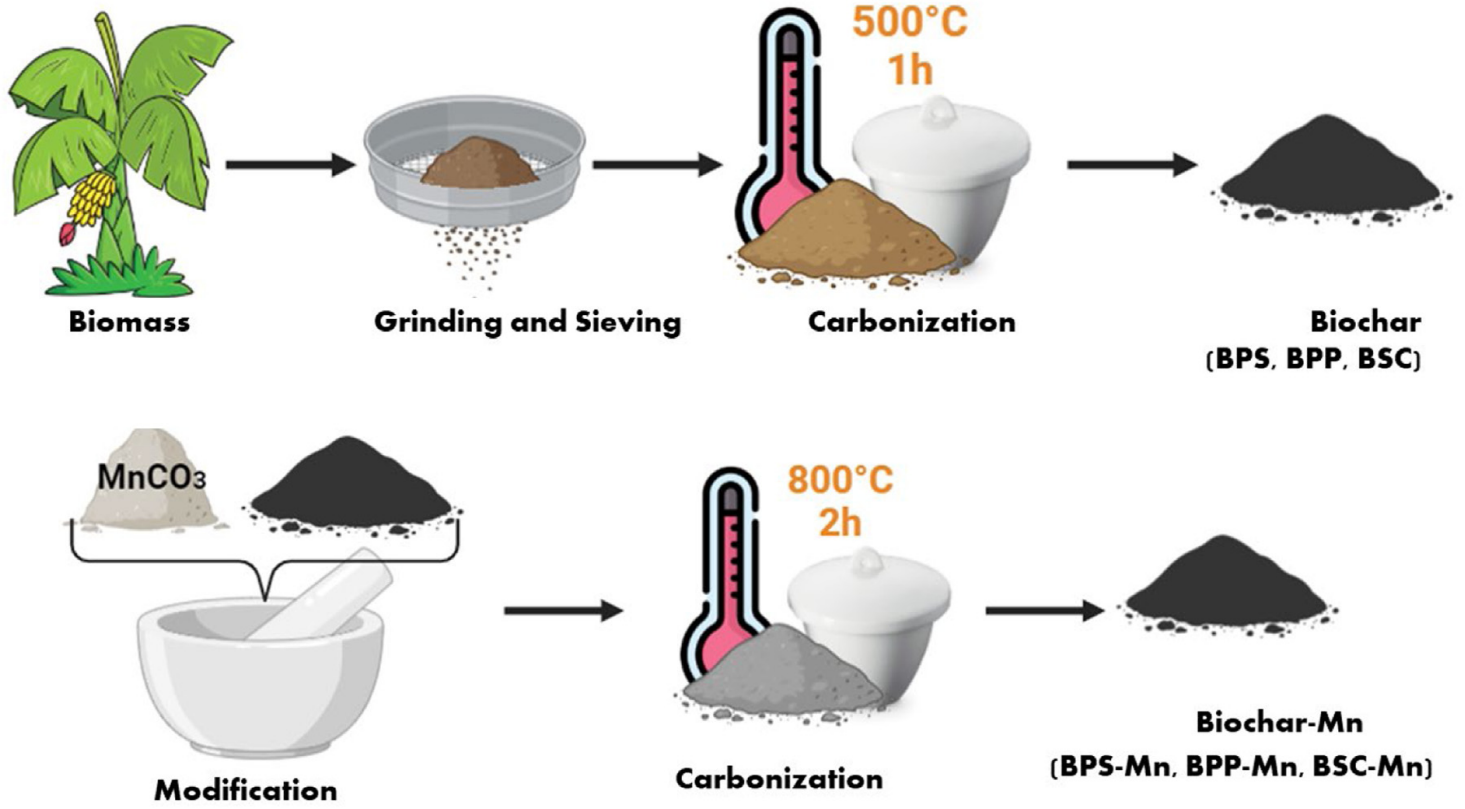
Method for synthesized carbonaceous material modified with manganese carbonate to promote the formation of singlet oxygen.

Six carbonaceous materials were prepared from the above-mentioned biomass, which were named as follows: BPS, BPP, and BSC. To prepare the materials, 40 g of the corresponding biomass was weighed and pyrolyzed for 2 h at 500 °C in a muffle furnace (Mueller & Krempel), obtaining the carbonized material or biochar (BPS, BPP, or BSC), and then modified with manganese carbonate (MnCO_3_) with an impregnation mass ratio of 1:1 pyrolyzed material/metal carbonate for 12 h. After impregnation, the material was heated at 800 °C for 1 h, obtaining our Mn-modified materials (BPS-Mn, BPP-Mn, and BCS-Mn). On the other hand, the pyrolysis process was carried at nitrogen atmosphere, with a flow rate of 124.8 mL min^−1^. Finally, carbonaceous materials were washed and dried at 105 °C for 24 h, Fig. 1.

**Spectroscopic and textural characterization of pyrolyzed and modified materials**

As is observed in Table 1, the presence of manganese significantly enhances the microporosity of the carbon-based materials. During carbonization or activation, these metals act as catalysts, modifying the carbon structure and increasing pore formation. Metals accelerate carbon gasification reactions, particularly when paired with activating agents like CO₂ or steam, which encourages the development of micropores and boosts surface area. Additionally, metal particles promote the rearrangement of carbon atoms, facilitating the removal of oxidized carbon and generating further micropores [9]. This process can be favoured in the presence of carbonate salts that contribute to the CO_2_ atmosphere. On the other hand, the surface area increases due to accelerated gasification reactions in the presence of metal salts, as well as the homogeneity [11] (Table 2).

**Table 1:** Textural properties of materials.

Material	Pore amount (%)		Hysteresis degree (%)	Surface Area S_BET_ (m^2^ g^–1^)
	Micropore	Mesopore		
**BPP-Mn**	87.0	13.0	3.5	264.9
**BPS-Mn**	79.8	20.2	3.6	252.1
**BSC-Mn**	82.0	18.0	2,9	170.1
**BPS**	38.5	61.5	3.8	17.0
**BPP**	14.6	84.4	6.2	17.3
**BSC**	23.8	76.2	23.0	11.1

**Table 2:** Surface properties and % Mn (EDS detection).

Material	%Mn	Zeta Potential
**BPP-Mn**	92.6	−11.0
**BPS-Mn**	95.8	−18.3
**BSC-Mn**	93.2	−16.3
**BPS**	5.5	−2.9
**BPP**	0.2	21.4
**BSC**	0	22.4

The presence of metals in carbon matrices alters the surface charge of materials. Electrostatic interactions between deprotonated oxygen groups on biochar and hydrogen bonds to electron-donating atoms of metal salts facilitate the formation of ionic compounds and coordination complexes. The high manganese content on the surface indicates that the metal has been incorporated. Additionally, negative zeta potential values suggest the presence of a metal carbonate salt in the system, confirming the coordination interactions.

## Evaluation of materials for generation of singlet oxygen

**Fig. 2:**
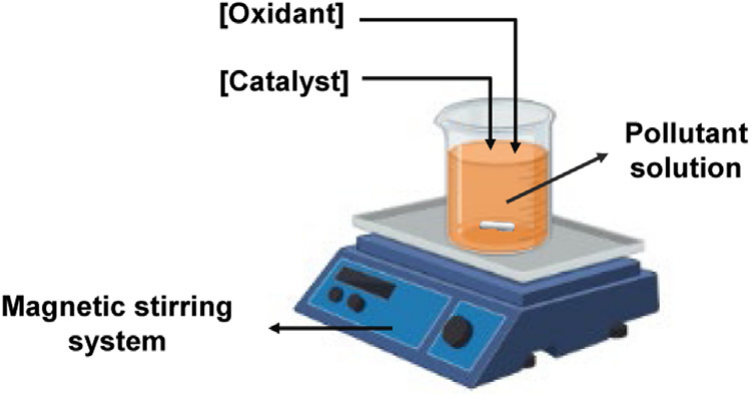
A reaction system is used to evaluate carbonaceous material to obtain a single oxygen.

To identify carbonaceous materials capable of activating the PMS for the removal of contaminants (ACE, SFM, and LOS), experiments were performed in a beaker with air circulation to promote an oxidizing atmosphere. with a magnetic stirring system. Scavengers were used to elucidate the participation of ROS in the degradation process. Methanol was used to quench $HO^{•}$ and${\;\text{SO}}_{4}^{•-}$, while sodium aside was utilized to determine the role of ^1^O_2_ species in the elimination of the contaminant, Fig. 2.

**Fig. 3:**
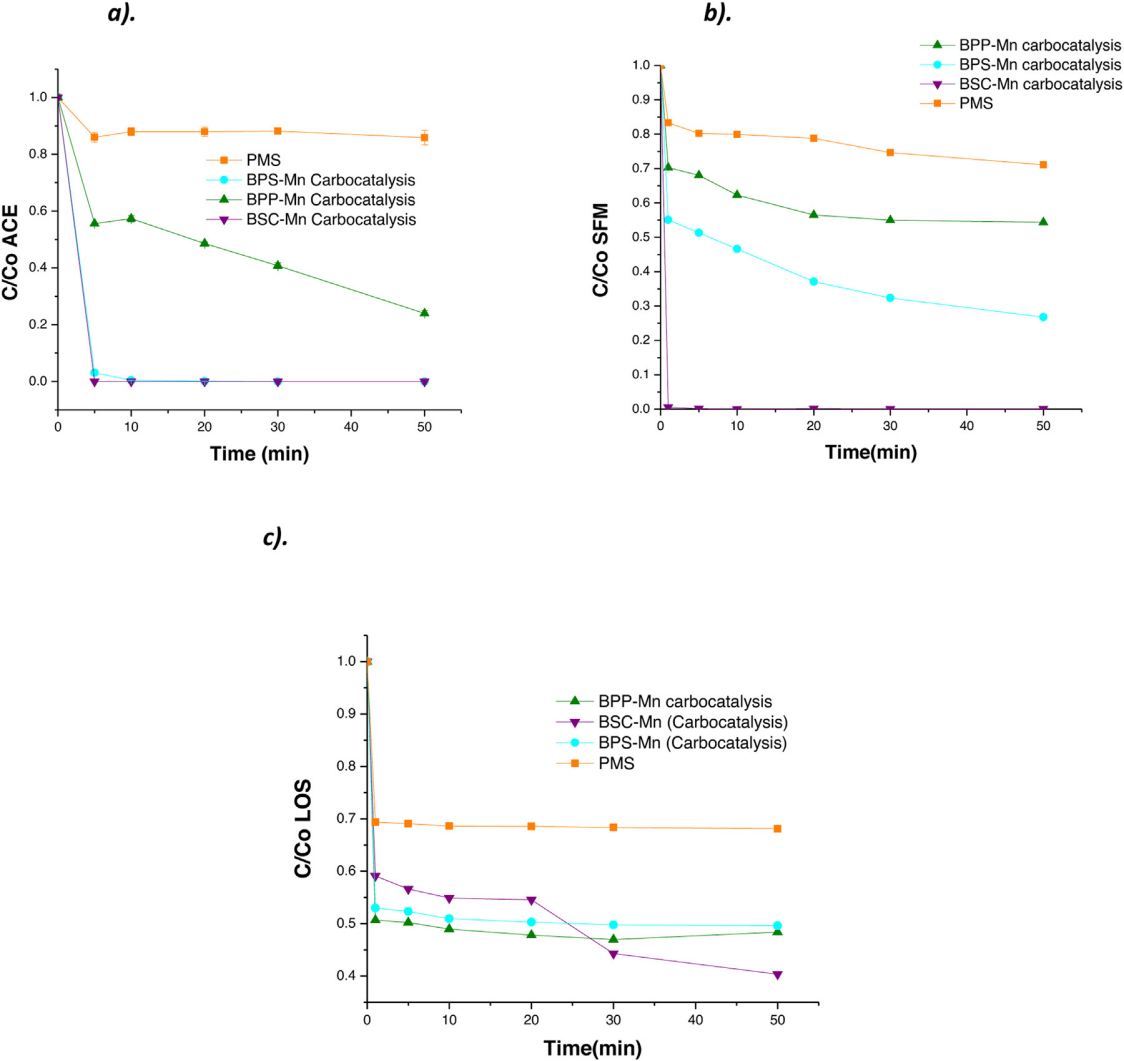
Kinetic process for the degradation of **a).** ACE, **b).** SFM and **c).** LOS using modified materials and PMS as a control.

**Fig. 4:**
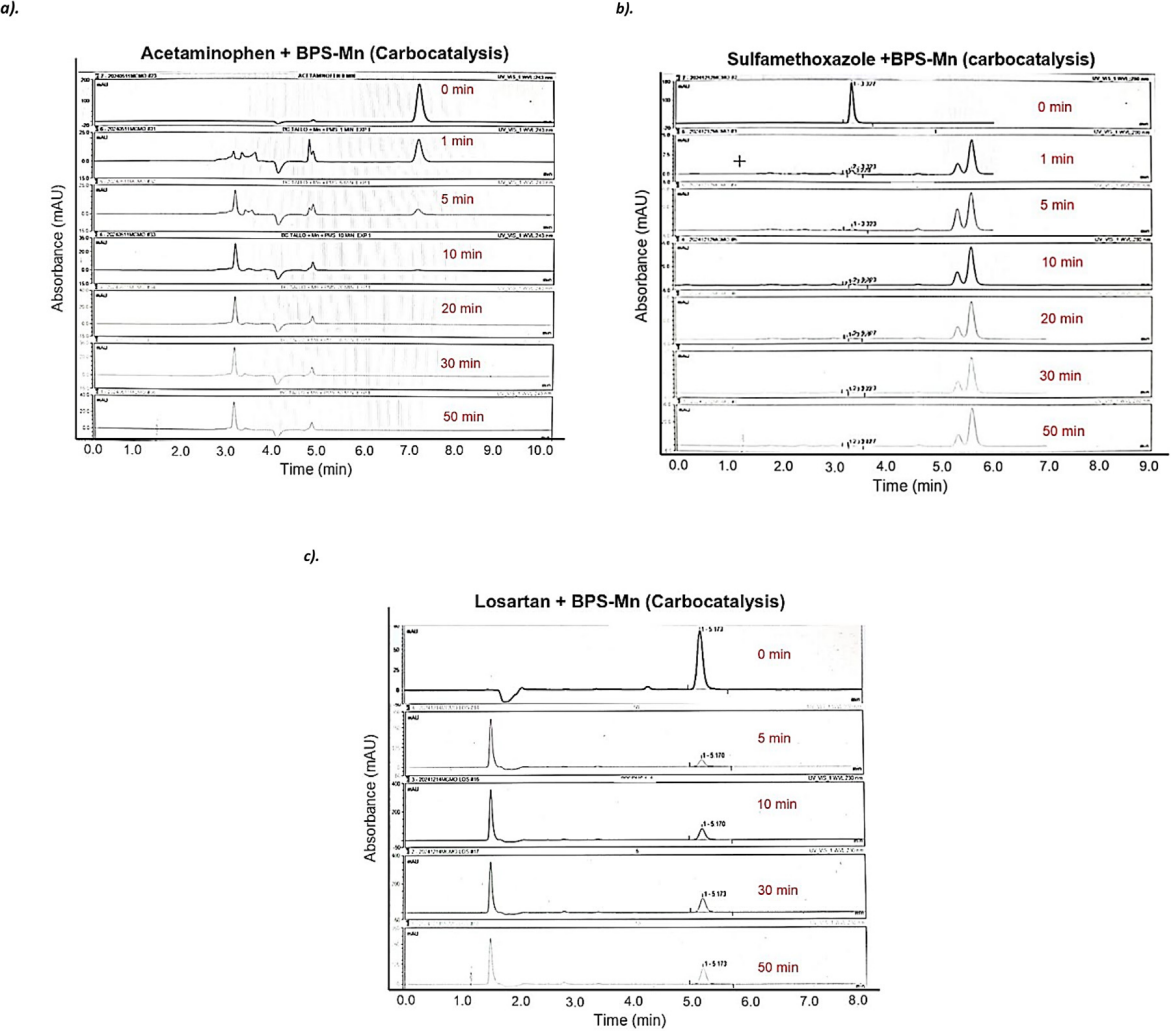
Kinetic process for the degradation of **a).** ACE, **b).** SFM and, **c).** LOS using modified materials and PMS as a control.

In Fig. 3, the materials BPS-Mn, BSC-Mn, and BPP-Mn demonstrate greater removal efficiency for ACE, SFM, and LOS compared to PMS when added on their own. Fig. 4 displays the chromatograms for the BPS-Mn material with the three molecules (ACE, SFM, and LOS) to distinguish whether the removal is due to adsorption or degradation processes. The results indicate that degradation products are formed for each molecule, confirming the occurrence of carbocatalysis.

**Fig. 5:**
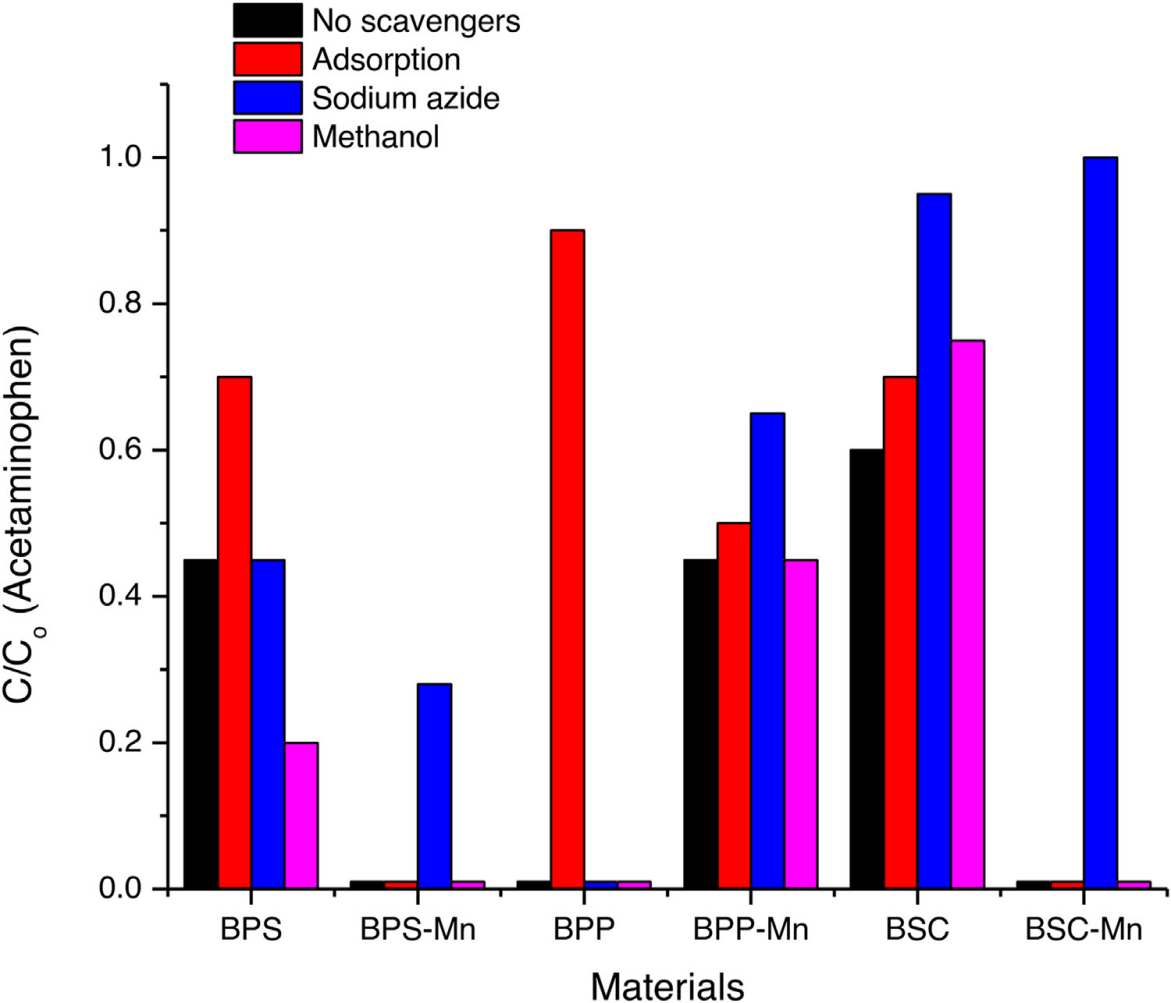
Elimination of ACE using pirolisized and modified materials in the presence and in the absence of scavengers for radical and unradical species (singlet oxygen: sodium aside, methanol: SO_4_^•−^ , ^•^OH. Acetaminophen (ACE, 30.6 µM), 0.5 mM of PMS, 0.2 g L^−1^ of the catalyst, scavenger 3000 µM.

As shown in Fig. 5, for a 20-minute cross-sectional treatment in each case (chosen for the observed differences in treatment results), it is evident that materials with only the pyrolyzed carbon matrix exhibit moderate adsorption of the acetaminophen molecule. However, they do exhibit different mechanisms associated with degradation, involving hydroxyl, sulfate radicals, and singlet oxygen. The ACE molecule is initially in a protonated, neutral state, which can promote π-stacking interactions, but the degradation percentage is lower than in the presence of manganese for the modified structures. The new materials exhibit different surface charge potentials, which can enhance adsorption and modify carbocatalysis. Likewise, as the reaction progresses, deprotonated molecules may settle on the material's surface, leading to changes in these properties. In contrast, pyrolyzed materials modified with manganese increase their interaction with ACE for BPS-Mn and BPP-Mn, which may be related to the surface charge potential of the materials, the pKa of ACE, and pH changes after treatment, as seen later. For singlet oxygen production, manganese-containing materials are significantly inhibited by sodium aside, which is consistent for this type of synthesis and demonstrates the selectivity of this material for producing this radical species compared to the pyrolyzed matrix alone. Both the mechanism of producing this species through the pentoxide anion radical and the HSO_5_⁻/SO_5_⁻ equilibrium can be favored by the presence of manganese in the structure—facilitating peroxide bond cleavage in the first case and deprotonation at basic pH in the second [12,13].

## Evaluation of pH in elimination ACE processes

The pH significantly influences the interaction between pollutants and the catalyst. Therefore, the pH carbocatalysis process was assessed before and after treatment. For modified materials from banana pseudostem and potato, the pH increased post-treatment. This increase suggests the presence of carbonate in the solution and the deprotonation of the ACE molecule, leading to the generation of singlet oxygen. With a pKa of approximately 9.4, PMS allows equilibrium between HSO_5_⁻ and SO_5_²⁻, which supports the presence of this non-radical species. Conversely, for BSC, a decrease in pH after treatment was observed, indicating a different mechanism. In acidic conditions, SO_5_•⁻ generation is favored, as shown in Eq. (4), Table 3.

**Table 3:** pH monitoring before and after ACE degradation.

Material	pH before	pH after
**BPP-Mn**	6.1	10.3
**BPS-Mn**	6.8	8.9
**BSC-Mn**	6.2	2.7
**BPS**	6.1	6.7
**BPP**	6.4	6.5
**BSC**	6.2	6.8

**Comprehensive the adsorption properties for biochar in BPS, BPP, and BSC in comparison biochar modified BPS-Mn, BPP-Mn, and BSC-Mn**

**Fig. 6:**
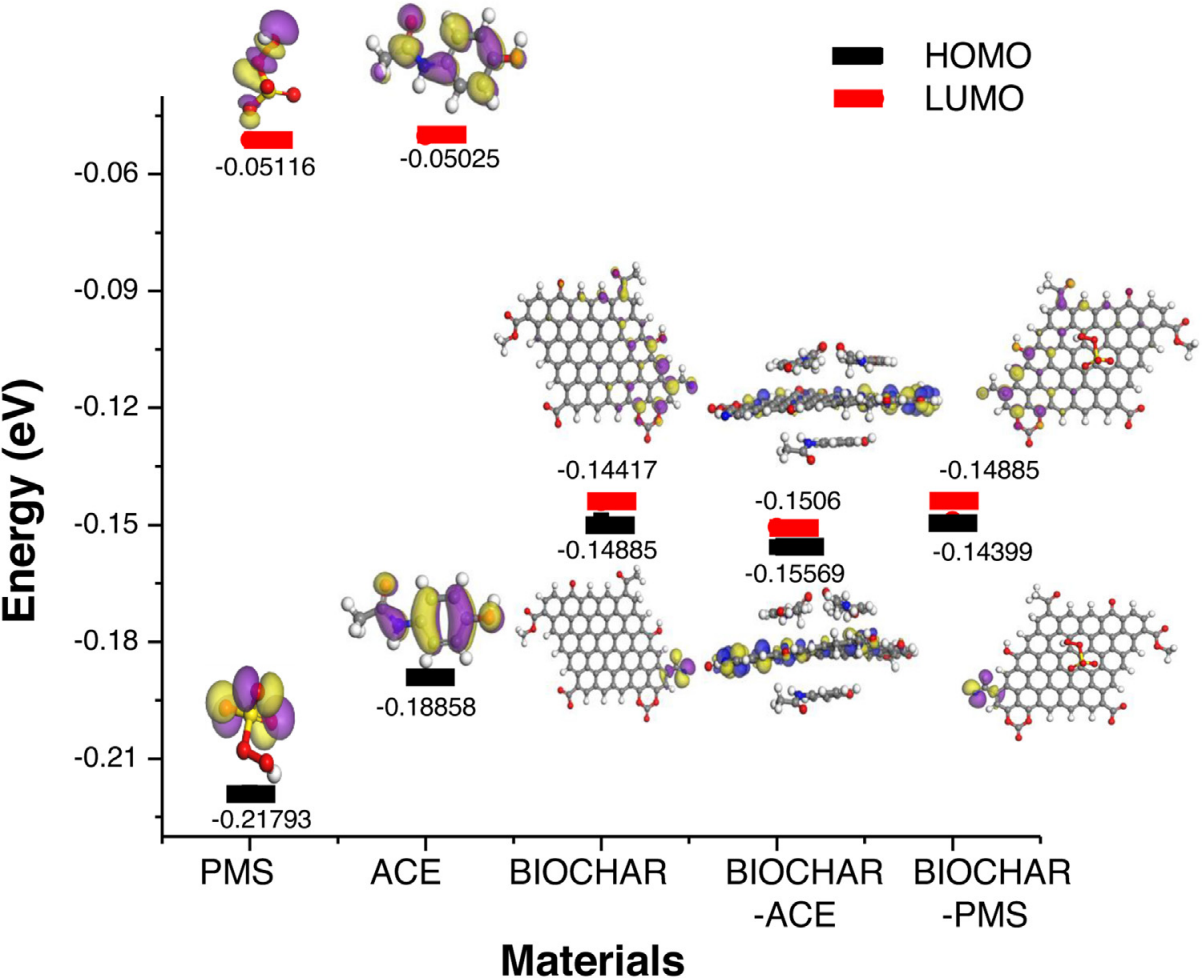
The optimized geometries and HOMO and LUMO energies for PMS, ACE, BIOCHAR, BIOCHAR-ACE, BIOCHAR- PMS were calculated with BIOVIA Material Studio 2017 with universal and Compass II forcefield.

The Materials Studio software BIOVIA 2019 19.1 was used to theoretically explore adsorption equilibria for a template of the pyrolyzed material and another for the modified material (target of BPS-Mn, BPP-Mn, BSC-Mn). The template was constructed using previously reported models for biochar, and the system was modified with a metal ion coordinated to functional groups in the biochar matrix. In the first case, it was observed that the minimum energy of the Biochar-PMS system exhibits a HOMO (Highest Occupied Molecular Orbital) state with higher energy than the Biochar-ACE system. This suggests that π-stacking interactions are more favorable between the molecule to be degraded and the carbon-based support. However, PMS molecules show lower adsorption to the carbon system, which could hinder its transfer to form singlet oxygen via PMS deprotonation. This indicates that degradation mechanisms via sulfate radicals, hydroxyl radicals, and singlet oxygen may be competing. Additionally, the system's energy further decreases with an increasing number of ACE molecules, optimizing at an interaction distance of 3 Å, yielding lower energy than the HOMO orbital corresponding to BIOCHAR-PMS; Fig. 6.

**Fig. 7:**
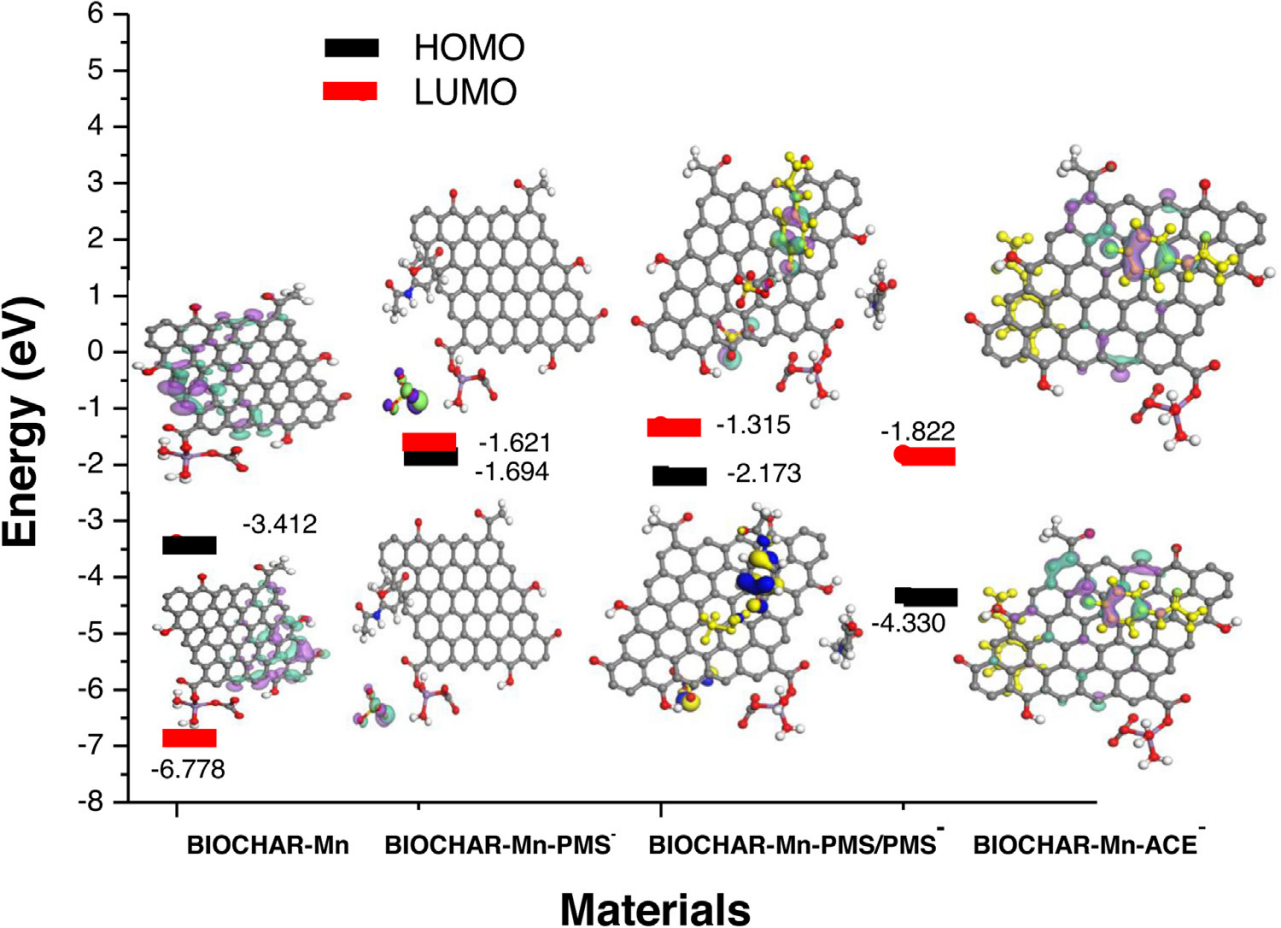
The optimized geometries and HOMO and LUMO energies for BIOCHAR-Mn, BIOCHAR-Mn-PMS deprotonated, BIOCHAR-Mn- (PMS-PMS deprotonated), BIOCHAR-Mn- ACE deprotonated) calculated with BIOVIA Material Studio 2017 with universal and Compass II forcefield.

As shown in Fig. 7, the interaction between manganese-modified biochar and ACE demonstrates lower energy (HOMO orbital) in equilibrium, which accounts for its high adsorption capacity. This interaction is located on the opposite side of the manganese carbonate presence, consistent with the negative charges of the metal salt and deprotonated ACE at high pH following treatment. On the other hand, the Mn-modified biochar in the presence of the (HSO_5_^−^/SO_5_^2−^) pair favors acetaminophen adsorption, aligning with rapid carbocatalysis in the presence of metal in the structure. Additionally, the (HSO_5_^−^/SO_5_^2−^) pair is observed in the same region, which promotes singlet oxygen formation. These results highlight the importance of system equilibrium under deprotonation conditions at a pKa of 9.4 for PMS. However, this proposal does not differentiate the other mechanism of singlet oxygen production—via the SO_5_^•−^ radical at circumneutral pH—based on the template condition of BSC-Mn, SO_5_^•−^.

The final remarks are:

• It is found that the presence of singlet oxygen produced by the interaction of carbon matrices with manganese carbonate and peroxymonosulfate is consistent.
• The pH of the solution at the end of treatment suggests the process via deprotonation of PMS-/PMS, whereas a low pH suggests the presence of the pentoxide radical anion, which releases *H*^+^ in solution when interacting with oxygenated groups of the carbon matrix.
• Regarding the unmodified biochar without metal, it is observed that the pH during treatment is circumneutral, and although this leads to carbocatalysis, the production of other oxidizing species such as sulfate radical anion and hydroxyl radicals stabilizes, competing with singlet oxygen.
• This is a promising method to promote the production of singlet oxygen and degrade emerging molecules such as acetaminophen.

## Limitations

The limitations of this method include its application in real water settings, as its effectiveness has yet to be explored in complex industrial, hospital, or wastewater environments where organic molecule mixtures could alter interactions with the carbon matrix. However, this method provides an excellent foundation for designing new carbonaceous materials aimed at selective catalysis, directed by singlet oxygen rather than more oxidative, short-lived species like hydroxyl radicals.

## CRediT authorship contribution statement

**Camila Mosquera-Olano:** Methodology, Data curation, Investigation, Writing – review & editing. **Carolina Quimbaya:** Methodology, Data curation, Investigation. **Santiago López-Pérez:** Methodology, Data curation. **Elkin Castellón-Castrillón:** Data curation. **Sandra Navarro:** Data curation. **John Rojas:** Methodology, Data curation, Investigation, Writing – review & editing. **Jorge Acosta:** Methodology, Data curation, Investigation. **Ricardo A. Torres-Palma:** Conceptualization, Methodology, Data curation, Investigation. **Yenny P. Ávila-Torres:** Conceptualization, Methodology, Investigation, Funding acquisition, Resources, Writing – review & editing.

## Declaration of competing interest

The authors declare that they have no known competing financial interests or personal relationships that could have appeared to influence the work reported in this paper.

## Data Availability

No data was used for the research described in the article.

## References

[1] Xiao S. (Mar. 2020). Iron-mediated activation of persulfate and peroxymonosulfate in both homogeneous and heterogeneous ways: a review. Chem. Eng. J..

[2] R. Ahmad, “Introductory chapter: reactive oxygen species—Origin and significance,” 2024. doi: 10.5772/intechopen.114146.

[3] Humayun S., Hayyan M., Alias Y. (Jan. 2025). A review on reactive oxygen species-induced mechanism pathways of pharmaceutical waste degradation: acetaminophen as a drug waste model. J. Environ. Sci..

[4] Clennan E.L., Pace A. (Jul. 2005). Advances in singlet oxygen chemistry. Tetrahedron.

[5] Zhu S., Li X., Kang J., Duan X., Wang S. (Jan. 2019). Persulfate activation on crystallographic manganese oxides: mechanism of singlet oxygen evolution for nonradical selective degradation of aqueous contaminants. Environ. Sci. Technol..

[6] Guo R. (Feb. 2024). A new generation pathway of singlet oxygen in heterogeneous single–atom Mn catalyst/peroxymonosulfate system. Chem. Eng. J..

[7] Yang Z., Yang X., Zhang W., Wang D. (Aug. 2024). Asymmetrically coordinated Mn-S1N3 configuration induces localized electric field-driven peroxymonosulfate activation for remarkably efficient generation of 1O2. Small.

[8] Quimbaya-Ñañez C., Serna-Galvis E.A., Silva-Agredo J., Huerta L., Torres-Palma R.A., Ávila-Torres Y. (Apr. 2024). Mn-based material derived from industrial sawdust for the elimination of ciprofloxacin: loss of antibiotic activity and toxicity via carbocatalysis assisted by ultrasound. J. Environ. Chem. Eng..

[9] Kohantorabi M., Moussavi G., Giannakis S. (May 2021). A review of the innovations in metal- and carbon-based catalysts explored for heterogeneous peroxymonosulfate (PMS) activation, with focus on radical vs. non-radical degradation pathways of organic contaminants. Chem. Eng. J..

[10] Romero-Hernandez J.J., Paredes-Laverde M., Silva-Agredo J., Mercado D.F., Ávila-Torres Y., Torres-Palma R.A. (Jan. 2024). Pharmaceutical adsorption on NaOH-treated rice husk-based activated carbons: kinetics, thermodynamics, and mechanisms. J. Clean Prod..

[11] Kumar K. (Dec. 2023). Biomass waste-derived carbon materials for sustainable remediation of polluted environment: a comprehensive review. Chemosphere.

[12] Zhou Y. (Nov. 2017). Activation of peroxymonosulfate by phenols: important role of quinone intermediates and involvement of singlet oxygen. Water Res..

[13] Miyoshi N., Tomita G. (Feb. 1979). Quenching of singlet oxygen by sodium aside in reversed micellar systems. Zeitschrift für Naturforschung B.

